# Nutritional Counseling in Survivors of Childhood Cancer: An Essential Component of Survivorship Care

**DOI:** 10.3390/children1020107

**Published:** 2014-08-14

**Authors:** Elena J. Ladas

**Affiliations:** 1 Division of Pediatric Hematology/Oncology/Stem Cell Transplant Columbia University Medical Center, 161 Ft. Washington, New York, NY 10032, USA; E-Mail: ejd14@cumc.columbia.edu; Tel.: +1-212-305-7835; Fax: +1-212-305-5848; 2 Institute of Human Nutrition, Columbia University Medical Center, 630 West 168th St., #1512. New York, NY 10032, USA

**Keywords:** nutrition, dietary intake, pediatric oncology, survivorship, dietary guidelines

## Abstract

There is a growing body of evidence suggesting that nutritional status during treatment for cancer has a significant impact on treatment-related toxicities and outcomes among children and adolescents with cancer. The effects of nutritional status appear to extend into survivorship with a large proportion of survivors at risk for a variety of nutrition-related morbidities. The influence of dietary intake on overall treatment outcomes and long-term morbidities is largely unknown. In adults, evidence suggests that greater adherence to cancer prevention dietary guidelines improves long-term health outcomes among survivors of cancer. Surveys describing dietary intake among survivors of childhood cancer have found that most survivors are not meeting the recommended guidelines for many dietary nutrients and this may have an unfavorable effect on nutrition-related outcomes. However, more research is needed in this area so that well-designed clinical trials may be developed and tested. This review presents an overview of the existing literature describing dietary intake among survivors of childhood cancer, the clinical implications of reported dietary behaviors among survivors, and identifies areas for future research.

## 1. Introduction

In 1981, Doll and Peto published a landmark paper that estimated that nearly 70% of cancer risk is attributable to diet and exercise; 35% of cancer risk may be attributed to diet alone [1]. This paper ignited a series of cohort studies that explored the association between dietary intake of food groups or select nutrients and risk of cancer, primarily among adult populations [2]. This research has led to the general consensus that following a cancer prevention diet that is high in fruits and vegetables, complex whole grains, and lean proteins may reduce the risk of select cancers. An increasing number of clinical studies are endorsing cancer prevention dietary guidelines and determining that adherence to these guidelines leads to clinically meaningful outcomes after a cancer diagnosis. For example, a study among 2000 adult female survivors of cancer found that adherence to cancer prevention dietary guidelines is associated with lower all-cause mortality (HR = 0.67; 95% confidence of interval (CI), 0.50–0.94) [3]. A similar finding was reported in one of the largest, ongoing cohort studies performed among nearly 400,000 adults, the European Prospective Investigation into Cancer and Nutrition study. A recent report from this study found that death from chronic disease and cancer was found to be 34% lower among those who adhered to a cancer prevention diet compared to survivors with lower adherence [4]. These results are encouraging and underscore the importance of providing nutritional counseling that is directed at dietary risk factors associated with the risk of death or chronic disease.

Success in the treatment of childhood cancer has led to estimates that 1 in every 250 adults are a survivor of a pediatric malignancy [5]. Despite these significant gains in survival, a growing number of studies have reported that up to 62% of survivors experience at least one chronic health condition; such as, obesity, metabolic syndrome, and cardiovascular disease [6]. While the development of chronic diseases among survivors of childhood cancer may be largely attributed to the treatment itself [6], the role of dietary behaviors in accelerating the development of nutrition-related conditions has garnered increased scientific scrutiny [7]. Much of the existing nutrition literature has been limited to anthropometric measures of nutritional status and have described the association between obesity and chronic disease, primarily among survivors of acute lymphoblastic leukemia (ALL). Most recently, Zhang *et al*. performed a meta-analysis of weight status among survivors of ALL and found that survivors’ mean BMI percentile is approximately 80%, a figure significantly higher than the general pediatric population [8]. Clinical efforts aimed at reducing the prevalence of obesity among survivors will likely necessitate combined medical approaches that include tailored, nutritional interventions. Clinical guidelines developed from scientific evidence, rather than expert opinion, will encourage the inclusion of dietary counseling into survivorship care [8,9,10].

Independent of weight, unhealthy dietary behaviors have been associated with increased risk for chronic health conditions such as heart disease and diabetes among pediatric and adolescent populations. Considering the pre-existing risk factors facing survivors of childhood cancer, the effect of unhealthy dietary behaviors is likely to be more pronounced among this population. Thus, an understanding of existing dietary behaviors among survivors of childhood cancer becomes an essential first step in order to develop effective nutritional interventions.

## 2. Dietary Intakes Reported by Survivors of Childhood Cancer

Table 1 presents a summary of select published studies reporting on dietary intake among survivors of childhood cancer. One of the first studies to describe dietary patterns among survivors was a cross-sectional study performed among 209 survivors of childhood cancer [11]. Responses were provided on existing diet and exercise behaviors, as well as, survivors’ interest and confidence in adopting healthy dietary behaviors. The investigators found that the majority of survivors were not meeting fruit and vegetable guidelines (79%) and only 16% were following a low-fat diet, a figure above that reported in both U.S. and Canadian healthy children [12,13]. Importantly, this article found that a significant barrier to increasing consumption of fruits and vegetables was confidence in one’s ability to adopt healthier behaviors. A subsequent cross-sectional study performed among 72 survivors of ALL reported similar findings [14]. In comparison to three commonly used dietary guidelines, no participant reported adherence to any combination of dietary patterns. Survivors reported low consumption of fruit, vegetables, and whole grains and high consumption of dietary sodium and added sugar intake, known risk factors for multiple chronic diseases. Others have confirmed these findings with the majority of studies reporting that less than half of the survivor population is meeting the recommended guidelines for fruit and vegetables, fiber, or dietary fat [15,16,17,18,19,20]. In healthy children, between 78%–90% of children are below the recommended intakes for fruit and vegetables, figures that are within or below those reported among survivors of childhood cancer [21].

A smaller number of studies have reported on select micronutrients, primarily vitamin D and calcium among survivors of ALL. Tylavsky *et al*. reported that less than 30% of survivors met dietary intakes for bone-metabolizing nutrients, a finding lower than reported in the general population [22]. For examples, Salvo, *et al*. reported that 95% of Hispanic children and 97% of African-America were below the RDA for vitamin D and 31% and 38% were below the RDA for calcium, respectively [23]. Low intakes of vitamin D and calcium may not be limited to survivors of ALL; similar findings were reported in other cross-sectional surveys [24]. Mays *et al*. reported that dietary intake of food sources rich in calcium was associated with readiness to change behavior, underscoring the importance of the inclusion of behavioral theories in nutritional interventions [25]. To date, there is limited information on dietary intake of other micronutrients among survivors of childhood cancer. Of the available studies, most report that survivors are meeting dietary intakes for most B vitamins, vitamin A, and other micronutrients [16,22]

**Table 1:** Summary of select studies reporting on dietary intake in survivors of childhood cancer.

Author/Year	Demographics	Design		Main Dietary Findings
Badr *et al*.; 2013 [29]	N = 170 /% M: 52 Mean age: 17.7 years Diagnosis: Mixed	Cross-sectional survey		96% below guidelines for fiber. 76% below guidelines for fruit and vegetables. 39% not meeting guidelines for dietary fat. Survivors who did not meet guidelines for fat intake experienced significantly ↑ general fatigue (*p* = 0.04) and cognitive fatigue (*p* = 0.04) compared to those who met guidelines. More males met guidelines for fiber (*p* < 0.0001) and fruit and vegetables (*p* < 0.01) compared to females.
Butterfield, *et al*.; 2004 [20]	N = 541/% M: 54 Mean age: 30.7 years Diagnosis: Mixed	Cross-sectional survey		68% eating more than recommended amount of red meat
Cohen *et al*.; 2012 [16]	N = 50/% M: 60 Mean age: 7.12 years Diagnosis: Mixed	Cross-sectional survey		54% of survivors exceeded estimated energy requirements by at least 110% For folate, calcium, and iron: 50%, 32%, 44% were below recommendations, respectively Most met recommendations for protein, thiamin, riboflavin, niacin, vitamin C, vitamin A, magnesium, phosphorous, and zinc.
Demark-Wahnefried *et al*.; 2005 [11]	N = 209/% M: 50 Mean age: 20.3 years Diagnosis: Mixed	Cross-sectional survey		79% below guidelines for daily fruit. 68% below guidelines for calcium 84% below guidelines for dietary fat intake
Landy *et al*.; 2013 [17]	N = 91/% M: 46 Mean age: 19 years Diagnosis: Mixed	Cross-sectional survey		30% exceeded recommended values for total caloric intake; however, similar to sibling controls. ↓ total HEI scores associated with ↑ % body fat (β = −0.19, *p* = 0.04). Mean HEI score was 55.5; survivor diets moderately adherent to recommendations and dietary quality similar to siblings. Survivors exposed to cranial irradiation had lower total HEI scores (−6.4, *p* = 0.01). HEI scores were lowest for dark green and leafy greens, whole fruits, and whole grains.
Love *et al*.; 2011 [18]	N = 102/% M: 46 Mean age: 14.3 years Diagnosis: ALL	Cross-sectional survey		Normal weight survivors consumed an average of 2364 kcal/day, 315 g carbohydrate/day, 91 g protein/day, and 84 g fat/day Overweight survivors consumed an average of 2472 kcal/day, 320 g carb/day. 106 g protein/day, and 88 g fat/day
Mays *et al*.: 2012 [25]	N = 75/% M: 48 Mean age: 14.2 years Diagnosis: Mixed	Randomized, controlled trial		Survivors classified as “readiness to change” consumed significantly more milk compared to those classified as “no readiness to change” (*p* < 0.001). Survivors classified as “readiness to change” were also more likely to meet calcium recommendations (*p* = 0.01) and consume increased milligrams of calcium (*p* = 0.006).
Mulhern *et al*.; 1995 [33]	N = 40/% M: 55 Survivor age range: 11–29 years Diagnosis: Mixed	Cross sectional survey	Respondents perception of diet quality (pre-adolescence/adolescent and young adults): 9%/2.5% never eating balanced meals; 4.6%/5% seldom eating balanced meals; 19.1%/27.5% sometimes eating balanced meals; 58.2%/47.5% most times eating balanced meals ; 16.4%/17.5% always eating balanced meals Dietary behaviors were positively correlated with other health-related behaviors.	
Phillips-Salimi *et al*.; 2012 [35]	N = 651/% M: 19 Mean age: 33 years Diagnosis: Mixed	Cross-sectional survey	75% below recommendations for fruits and vegetables, which was not significantly different to controls.	
Reeves *et al*.; 2007 [15]	N = 28/% M: 39 Mean age: 21 years Diagnosis: Mixed	Cross-sectional survey	79% below guidelines for fruit 96% below guidelines for vegetables	
Robien *et al*.; 2008 [14]	N = 72/% M: 42 Mean age: 29.9 years Diagnosis: ALL	Cross-sectional survey	Adherence to any set of guidelines was not reported by any participant. 50% below guidelines for fruits and vegetables and dietary fat. Sodium and added sugar consumed in considerable excess of recommendations and whole grains in levels below recommendations. For the DASH and USDA Food Guide Scores, women received higher scores compared to men. For WCF/AICR Cancer Prevention Score, men received significantly higher scores compared to females (*p* < 0.01).	
Tyc *et al*.; 2001 [19]	N = 46/% M: 61 Mean age: 13.6 years Diagnosis: Mixed	Cross-sectional survey	Respondents perception of diet quality: 15% never or seldom eating balanced meals 22% sometimes eating balanced meals 47% most times eating balanced meals 15% always eating balanced meals	
Tylavsky *et al.* [22]	N = 164/% M: Mean age: 19.6 years Diagnosis: ALL	Cross-sectional survey	Average HEI was 58.9 (<19 years) and 56.9 (>19 years) 60% exceeded guidelines for fat. <30% of participants met recommended intakes for vitamin D, calcium, potassium or magnesium. For folate and vitamin E, 48% and 77% were below guidelines, respectively. Most survivors met guidelines for vitamins A, B6, B12, C, thiamin, and riboflavin. Energy intake from sweets 70% above recommended values.	

## 3. Barriers to Healthy Dietary Behaviors

It has been well-established that health behaviors are highly complex and embedded in larger psychosocial, environmental and economic contexts [26]. The finding that a large proportion of survivors are not meeting recommended intakes for most nutrient categories has driven the need to understand barriers to and perceptions of adopting healthy dietary behaviors. Arroyave *et al*. conducted a cross-sectional survey in 118 survivors between the ages of 13–35 on reasons for low dietary intake of fruits and vegetables, whole-grains, and low-fat foods [27]. Most commonly, respondents attributed poor consumption of healthy dietary items to availability, taste, cost, satiation, and either social or family influences. Another small, qualitative survey performed among 20 young adult survivors found that respondents were interested in healthier dietary options but that difficulty finding information, receiving confusing or conflicting information, along with the stress associated with making nutritional decisions and weight-loss programs prevented changes in dietary intake [28]. Other studies have also reported a strong interest among survivors in learning about healthy dietary habits. A cross-sectional survey that obtained information on interest in dietary program from 170 survivors with a 5-point Likert scale (0 = extremely interested to 5 = not at all interested). The survey found that 75% were “very” or “extremely” interested in weight loss programs whereas 84% were interested in learning to eat more nutritiously [29]. However, survivors have reported that health professionals do not uniformly recommend or endorse the importance of dietary change for survivors. For example, one survey performed among adult survivors of cancer reported that respondents reported that a mere 16% were advised by their physician to increase consumption of fruits and vegetables and 29% reported physician recommended a low-fat diet. This may reduce the urgency for patients to allocate resources and time to changing existing dietary behaviors, [30] thus nutritional education and research may need to improve the delivery of nutritional information to health care providers during and after treatment for cancer.

## 4. Clinical Implications for Survivors

The clinical implications of unhealthy diets on the risk of cancer among adolescents are limited and focus primarily on dietary intake during adolescence and risk of breast cancer [31]. Most of these studies describe dietary intake of single nutrients or food groups rather than exploring comprehensive indices of dietary intake (e.g., Healthy Eating Index). Among survivors of childhood cancer, preliminary studies suggest that better quality diets may result in significantly improved clinical outcomes in the long-term, but the association of dietary intake and risk of relapse or cancer has not been reported. In a cross-sectional study among 170 survivors of childhood cancer, improved adherence to national dietary guidelines was significantly associated with improved quality of life and reduced fatigue [29]. Moreover, this study identified at-risk groups of survivors who may be more vulnerable to the effects of suboptimal dietary practices. The authors reported that females, adolescence and young adults (AYA), and survivors of tumors of the central nervous system or lymphoma may be at higher risk of following poor dietary patterns compared to other groups of survivors. Tonorezos *et al*. reported that with greater adherence to the Mediterranean Diet, the odds of developing metabolic syndrome fell by 31% (OR 0.69; 95% CI 0.50, 0.94; *p* = 0.019) [32], lending further support for the promotion of anticancer dietary behaviors in survivors of childhood cancer. Other cross-sectional surveys have reported positive associations between adherence to dietary recommendations and a higher frequency of physical activity suggesting that healthy dietary behaviors may promote the adoption of other healthy lifestyle behaviors [33].

One study explored the effect of supplementation with and without dietary counseling on the D in survivors of ALL [34]. Participants were randomized to nutritional counseling with or without supplementation that consisted of calcium and vitamin for a 2 year period. The authors found that the addition of supplementation did not impart an added benefit over and above that of dietary counseling alone, further lending support to the need for the provision of nutritional counseling to survivors. It is plausible that remediation of low dietary intakes through supplementation does not offset the benefits of comprehensive nutritional counseling. However, this study has several limitations including a wide age range among study participants reflecting different stages of bone maturation and a high-drop out rate, potentially introducing bias to the study findings.

Taken together, the limited data suggest that survivors of childhood cancer are not adhering to cancer prevention guidelines and are engaging in lifestyle behaviors that may further increase the risk for the development of nutrition-related chronic conditions. Effective dietary interventions may impact a large proportion of chronic conditions faced by survivors of childhood cancer, thus significantly altering the quality life after completion of cancer treatment. Phillips-Salimi *et al*. reported on health behaviors and late-effects after cancer treatment [35]. Of the eight evaluated late-effects, diabetes, high blood pressure, high cholesterol, heart attack, angina/coronary heart disease, stroke, asthma, and arthritis, four may be moderated through effective dietary interventions. The finding that dietary behavior is associated with other healthy behaviors; such as physical activity, suggests that interventions targeting dietary strategies may positively impact other healthy behaviors among survivors potentially expanding the impact of dietary change on long-term health outcomes.

## 5. Current Dietary Guidelines for Survivors

Clinical guidelines are an important component of optimizing the delivery of cancer care and have contributed to the advances in other aspects of supportive care within pediatric oncology [36,37]. Dietary guidelines serve to guide clinicians in providing reliable nutritional information to patients and their families and provide families with a trustworthy resource to obtain nutritional information. The importance of adhering to comprehensive cancer-prevention guidelines appears to have a clinical impact among survivors of adult cancer. A recent meta-analysis reported that adults that adhere to the recommended cancer-prevention guidelines experienced a 21% reduction in risk of colon cancer and 22% reduction in cancer-specific risk [38].

In pediatric oncology, most of the existing nutritional guidelines were developed by expert consensus and grounded in data collected among adults. Two of the most widely referred to guidelines designed for survivors of cancer are the American Cancer Society [39] and the World Cancer Fund/American Institute for Cancer Research [40]; however, neither address survivors of childhood cancer. The Children’s Oncology Group (COG) [41] has designed nutritional guidelines as part of survivorship care; yet, a survey of institutional practices in COG suggest there is limited application of these guidelines into existing clinical care models [9]. In general, these guidelines are similar to those of the American Cancer Society in that diets should emphasize fruits and vegetables, whole grains, lean proteins, low-fat dairy products, and minimize dietary intake of processed meat. However, the impact of adhering to the recommended dietary behaviors on the development of late-effects related to cancer treatment or the development of recurrence/relapse or malignancies in adulthood is unknown.

## 6. Discussion and Recommendations for Research

With the developing evidence suggesting that dietary intake may alter the risk of nutrition-related chronic conditions and risk of cancer, it becomes essential to integrate dietary counseling into the clinical framework of survivorship care. Several priority areas may be identified from the existing literature to drive the development of high-quality clinical trials. First, more epidemiologic information is needed on the variability of dietary intake during and after treatment. It has been established that children who are overweight or obese experience reduced survival and are at increased risk of therapy-related toxicities [42,43,44]. Similar findings have been reported among adults with cancer such that excess weight is estimated to contribute to approximately 20% of cancer-related deaths [45]. The development of effective nutritional interventions may attenuate the effect of weight on outcomes; however, to accomplish this will require a greater understanding of the association of diet on previously reported health outcomes. Research of this nature must also identify if specified micronutrients or an array of dietary behaviors accelerate or prevent the development of nutrition-related morbidities. The identification of components of the diet that lead to the largest effect on clinically significant outcomes will direct the development of nutritional interventions. Ideally, these studies may be designed and tested through established research consortiums. A long-term goal of nutritional research in pediatric oncology must also encompass gene-nutrient interactions as emerging evidence suggest that an individual’s genetic make-up may have a significant role in the response to dietary interventions [46].

The association of dietary intake and late effects is likely to vary by diagnosis and therapeutic regimen. Future epidemiologic studies may consider expanding outside the context of ALL so that nutritional interventions may be modified and designed based on diagnosis and treatment exposures. This may be especially relevant for pediatric malignancies that constitute the growing number of survivors including Hodgkin’s Disease, acute myelogenous leukemia, and medulloblastoma. The optimal timing for nutritional interventions, as well as, the development of nutritional interventions that are adaptable to a wide range of ages, ethnicities, and socioeconomic groups are most likely to be easily woven into the survivorship model. Consideration may be given to initiating dietary counseling prior to entering survivorship care. Approaching survivorship with prevention-based interventions, may anticipate treatment-related late effects and result in reduced morbidities, improved quality of life, and reduced burden on the health care system.

Importantly, it is clear that education of clinicians providing care to survivors of childhood cancer is necessary to advance the provision of nutrition counseling to patients and their families. It is important that health messages be consistent and comprehensive [47]. There is a need for the development of nutritional algorithms, especially as few existing guidelines address the unique needs of survivors of childhood cancer. Ideally, these algorithms may provide clinical direction on dietary targets based upon existing dietary patterns, diagnosis, and treatment exposure. Nutritional committees within research consortiums are ideal forums to develop and test evidence-based nutritional guidelines for survivorship. Initiating nutritional counseling during or immediately after treatment within existing treatment protocols may further facilitate the delivery of nutritional information, create standardized and uniform recommendations, and provide investigators with the opportunity to evaluate the degree to which nutrition interventions improve clinical outcomes.

Finally, nutritional interventions must be comprehensive in their approach and multidisciplinary in nature. Nutritional interventions that encompass lifestyle-based approaches have been most effective in promoting long-term behavioral change in both the pediatric and adult populations [48]. Adapting successful interventions that have been performed in similar patient populations, yet address the unique needs of survivors, will likely yield the best clinical results among survivors of childhood cancer.
